# Supplementary material for: Photodimerization induced hierarchical and asymmetric iontronic micropatterns
Source: Nat Commun. 2022 Oct 30;13:6487. doi: 10.1038/s41467-022-34285-7 (PMC9618565; doi:10.1038/s41467-022-34285-7)
Supplement: Supplementary file 1 — Supplementary Information [file 41467_2022_34285_MOESM1_ESM.pdf]

SUPPLEMENTARY INFORMATION

Photodimerization Induced Hierarchical and Asymmetric Iontronic Micropatterns

Zehong Wang[1], Tiantian Li[1], Yixiang Chen[2], Jin Li[1], Xiaodong Ma[1], Jie Yin[1], Xuesong Jiang[1]*

[1] School of Chemistry & Chemical Engineering, Frontiers Science Center for Transformative Molecules, State Key Laboratory for Metal Matrix Composite Materials, Shanghai Jiao Tong University, Shanghai 200240, China

[2] Key Laboratory of Science & Technology of Eco-Textile, Ministry of Education. College of Chemistry, Chemical Engineering and Biotechnology, Donghua University, Shanghai 201620, China.

*Corresponding author: Xuesong Jiang, Ph.D. Professor, E-mail: ponygle@sjtu.edu.cn

Supplementary Methods

Materials

All Reagents were obtained from commercial sources and used as received without further purification, unless otherwise specified. 1-Vinylimidazole (VIm) and 1-Ethyl-3-methylimidazolium bis(trifluoromethylsulfonyl) imide ([EMIm][TFSI]) were purchased from Alfa Aesar. *n*-butyl acrylate (*n*-BA) was purchased from TCI America. 9-(Chloromethyl) anthracene was purchased from Meryer (Shanghai) Chemical Technology Co., Ltd. Bis (trifluoromethylsulfonyl) amine lithium salt was purchased from Bide Pharmatech Ltd. Azobisisobutyronitrile (AIBN) was purchased from Sigma-Aldrich and recrystallized from ethanol prior to use.

Characterizations

^{1}H NMR spectra were conducted on a Varian Mercury Plus500 MHz instrument with tetramethylsilane (TMS) as the internal standard and dimethylsulfoxide-d_6 (DMSO-d_6) as the solvents. FTIR spectrum was obtained by using a microscopic imaging infrared spectrometer (NICOLET IN10, Thermo). Average molecular weights were tested by means of gel permeation chromatography (GPC, LC-20A, Shimadzu, Japan) with N, N-Dimethylformamide acted as an eluent at a flow rate of 1.0 mL min^{-1}, and polystyrene standards using as the calibration curve. XRD spectra were measured on dropping coated iontronic films by X-ray diffractometer (D8 ADVANCE Da Vinci, Bruker, Germany). UV vis-spectra of dropping coated iontronic films on quartz glass was obtained by TU-1091 spectrophotometer (Persee, China). DSC results of iontronic films comprising of different molar content of IL were carried out by differential scanning calorimetry (DSC-2500, TA, American). Surface morphology observation was carried out by laser scanning confocal microscopy (LSCM, OLS5000, Olympus, Japan) and SEM (JEOL JSM-7800F Prime, Sweden). The measurement of surface conductivity was conducted by C-AFM (MFP-3D, Oxford Instrument, American) with Pt-coated tips. The interfacial capacitance measurements were obtained using an LCR meter (TH283X, Tektronix) at an AC excitation voltage of 500 mV and a sweeping frequency ranging from 20 Hz to 200 kHz. The surface resistivity measurements and the open-circuit voltage were carried out by a digital multimeter (Keithley 2110-220, Tektronix).

Synthesis of the anthracene-functionalized ionic liquid copolymer (An-PIL)

The precursor for the synthesis of An-PIL was prepared from the traditional free radical copolymerization of n-BA and VIm at the feed mole ratio of 2:1 (**Supplementary Fig. 1a**). First, 1-Vinylimidazole (3.76 g, 0,04 mol), n-BA (10.24 g, 0.08 mol) and recrystallized AIBN (0.14 g, 1 wt.% versus the total monomer weight) were dissolved in 1,4-Dioxane (30 mL) in a two-neck round-bottom flask. The solution was degassed using dry nitrogen for 30 min, filled

with dry nitrogen, immersed in an oil bath at 70 ºC, and maintained to react under vigorous mechanical stirring for 16 h. After cooling to room temperature, the polymer that denoted as P(VIm-BA) was precipitated in cold n-hexane, separatory filtration, dried at 60 ºC in a vacuum oven for 24 h to yield a transparent gel (12 g, yield: 85 %).

P(VIm-BA) (6.0 g, 17 mmol) was dissolved in DMF (60 mL) at 70 ºC. To this solution was added 9-(Chloromethyl) anthracene (3.89 g, 17 mmol) and the reaction mixture was maintained at 70 ºC for 18 h. After cooling the solution to room temperature, the polymer that denoted as P([AnVIm][Cl]-BA) was precipitate from cool diethyl ether and dried at 50 ºC in a vacuum oven for 12 h to yield pale brown solid (7.47 g, yield: 71%). (**Supplementary Fig. 1b**)

To a vigorous stirring solution of P([AnVIm][Cl]-BA) (4.2 g, 7.3 mmol) in DMF (40 mL) was added LiTFSI (2.30 g, 8 mmol) at room temperature. The final polymer that denoted as An-PIL was precipitated from DI water after 24 h, vacuum suction filtration, and dried at 50 ºC in a vacuum oven for 12 h to yield pale yellow solid (5.21 g, yield: 87 %). The resultant An-PIL has a number average molecular weight of Mn=11.4 kDa and polydispersity index (PDI) of 1.60. The different constituent ratio of the polymer is calculated by integrals in the ^{1}H NMR spectra. The peak above 6.0 corresponds to the signal of the proton connecting the anthracene and imidazolium. The signal below 1.0 corresponds to the proton connected to the methyl of butyl-acrylate. The actual constituent ratio in final copolymer An-PIL (x: y = 0.5:1) is approximately quantitative with the feed mole ratio. (**Supplementary Fig. 2**)

Supplementary Discussion

Mathematical Derivations for the compression-to-potential gradients

The piezoionic effect is resulted from Donnan like depolarization due to an inhomogeneous ionic distribution[1, 2]. The deformable HAIM initially at equilibrium and has a uniform distribution of mobile species, and the electrochemical potential experienced by all species is equal and the free energy of the system is minimal. When a mechanical compression causes the HAIM to non-homogeneously deform as shown in **Supplementary Fig. 11**, the ionic species will experience a differential pressure locally along with the polymeric bulk and result in gradient distributed charged ions. At this point, two kinds of ionic migration existed within HAIM[3], namely, i) compelled diffusion of cations from the stress-bearing surface (top side) to the bottom side and ii) the internal electric field-induced ionic drifting from the bottom side to the top side. This electrical potential gradient, ΔE, can be measured using external electrodes that are in contact with the material. Meanwhile, the compelled diffusion current density $j_{\mathrm{dif-i}}$ can be given by Supplementary Equation 1:

$$j_{\mathrm{dif-i}} = -z_i F D_i \frac{di}{dx} \ (1)$$

where F is Faraday constant, z_i, D_i, and i are the valence, diffusion coefficient, and ionic concentration of cation ith, respectively. Moreover, the drifting current density $j_{\mathrm{dri-i}}$can be represented as Supplementary Equation 2:

$$j_{\mathrm{dri-i}} = -z_i F i v_i \frac{dE}{dx} \ (2)$$

Among which, the mobility of cation i_{th} can be described as Supplementary Equation 3:

$$v_i = D_i \frac{z_i F}{RT} \ (3)$$

where, E is the self-induced potential of HAIM, R is the gas constant, and T is the temperature. The stable self-induced potential E of HAIM can be calculated when the diffusion and drifting effects reach a dynamic balance (i.e., $j_{\mathrm{dif-i}} = j_{\mathrm{dri-i}}$), which can be given by Supplementary Equation 4:

$$\mathrm{E} = \frac{RT}{z_i F} \mathrm{In} i \ (4)$$

we supposed that the cation ith was compelled to migrant and finally two locations separated by a distance Δx within the HAIM. Meanwhile, the $[i]_x$ and $[i]_{x+\Delta x}$ are concentrations of cation ith at different locations. The potential gradient of HAIM, ΔE can be calculated by Supplementary Equation 5:

$$\Delta E = \frac{RT}{ZF} \mathrm{In}\left(\frac{[i]_x}{[i]_{x+\Delta x}}\right) \ (5)$$

Modified bilayer model for gradient cross-linking layers of An-PIL film

According to our previous reports[4, 5], the buckling mechanism can be explained by a modified bilayer system that incorporates the UV-crosslinking-induced An-PIL gradient layer which contains the stiff surface and the soft substrate, as shown in **Supplementary Fig. 12**. The self-wrinkling (primary patterns) occurs as a result of minimizing the total energy stored in iontronic host, i.e. when the compressive strain (ε_0) at the surface exceeds a critical value (ε_c). The characteristic amplitude (A) of the wrinkles can be predicted on the basis of the intrinsic properties of the iontronic host involving the plane-strain moduli of the substrate ($\bar{E}_s$) and surface ($\bar{E}_f$), the surface thickness (h_f), and the applied compressive strain (ε_0) as in Supplementary Equation 6 and Equation 7:

$$\mathrm{A} = h_f \left(\frac{\varepsilon_0}{\varepsilon_c} - 1\right)^{1/2} \ (6)$$

$$\varepsilon_c = -\frac{1}{4}\left(\frac{\bar{E}_s}{\bar{E}_f}\right)^{2/3} \ (7)$$

Meanwhile, the UV light exposure region of the iontronic host was photo-crosslinked and resulting in a thermally induced biaxial compressive stress (ε_0) due to the mismatch in thermal expansion between the stiff surface (α_f) and soft substrate layers (α_s) during temperature

variation (ΔT). Heating, which may arise from the photodimerization reaction and the absorbed UV light energy, result in a gradient in the expansion of iontronic host. For a given thermally induced wrinkle system, the compressive strain can be estimated as in Supplementary Equation 8:

$$\varepsilon_0 = (\alpha_s - \alpha_f) \times \Delta T \ (8)$$

According to Eq.1 and Eq.2, when the parameters h_f and $\bar{E}_s$ are fixed, the modulus of the surface layer ($\bar{E}_f$) and the applied strain (ε_0) can be used as key parameters for the regulation of the self-wrinkling patterns. Since photodimerization crosslinking of iontronic host cause a stiff surface and higher surface modulus ($\bar{E}_f$) as well as lower thermal expansion coefficient of surface (α_f), the critical value (ε_c) was therefore decreased and the applied strain (ε_0) increased. Besides, due to the IL was a liquid solvent and thus enable the glass transition temperature (T_g) and crystallinity of iontronic host to be lower with the molar ratios of IL increased (Supplementary Fig. 6). Meanwhile, the modulus of substrate ($\bar{E}_s$) turn to be much lower and the thermal expansion coefficient of substrate (α_s) become larger along with more IL in iontronic host. Thus, the critical strain (ε_c) decreased significantly and also the applied strain (ε_0) increased dramatically with the IL molar ratios increase, meanwhile the amplitude continuously increased.

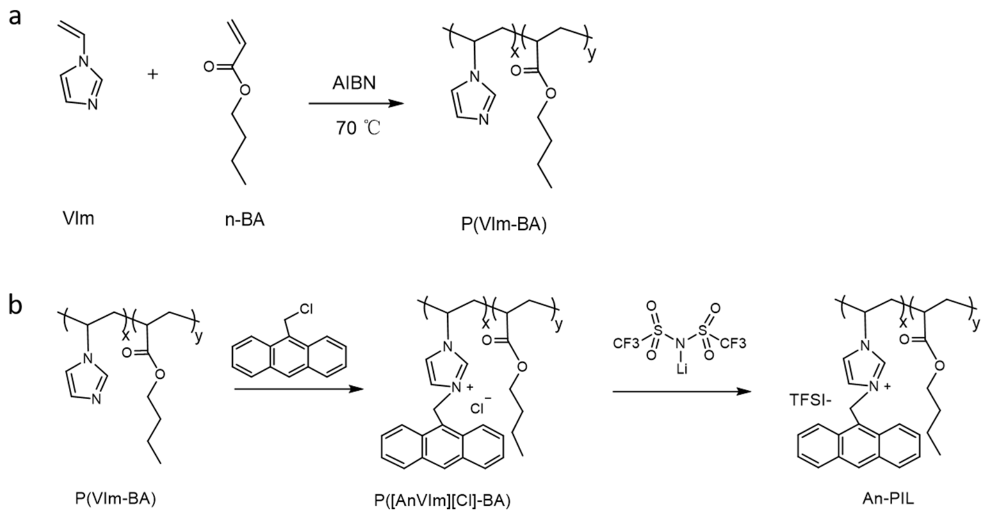

Supplementary Fig. 1. The synthesis of anthracene-functionalized ionic liquid copolymer (denoted as An-PIL).

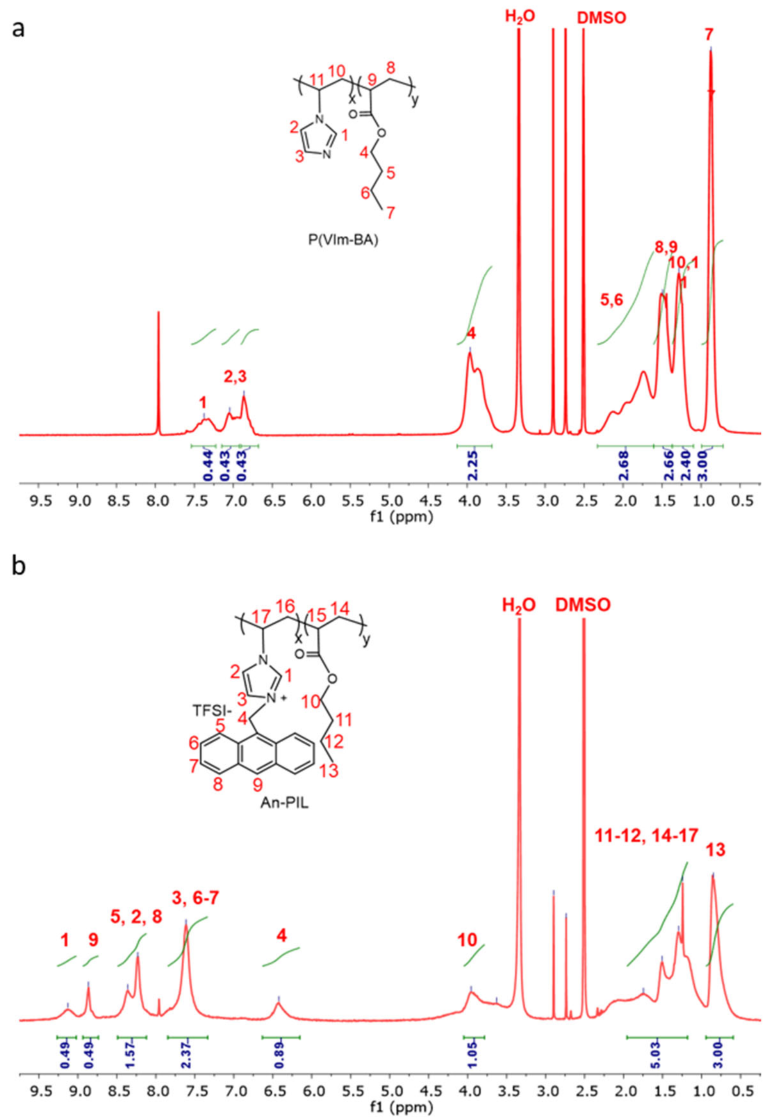

Supplementary Fig. 2. ^{1}H NMR spectra of **a** P(VIm-BA), and **b** An-PIL (i.e. the anthracene-functionalized ionic liquid copolymer, P([AnVIm][TFSI]-BA)) inDMSO-d_6 solution

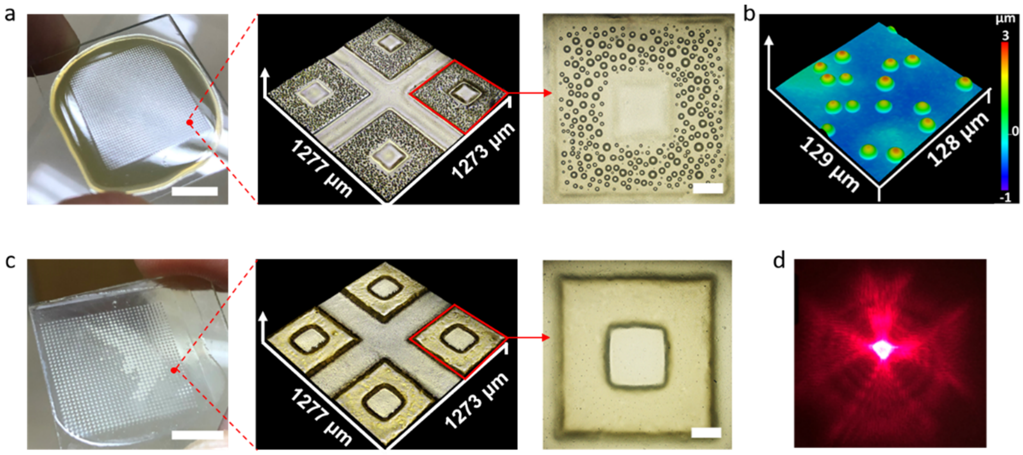

Supplementary Fig. 3. The resultant IL droplets @Primary patterns before and after washing with ethanol. a Optical images and the corresponding microscopy images of pristine IL droplets @primary patterns; scale bar: 1 cm for 1st picture, and 100 μm for 3rd picture. **b** The laser scanning confocal microscopy (LSCM) images of the IL droplets. **c** Optical images and the corresponding microscopy images of IL droplets @Primary patterns after removing IL droplets; scale bar: 1 cm for 1st picture, and 100 μm for 3rd picture. **d** The red light diffusion micropattern via using the resultant pure primary patterns as optical grating.

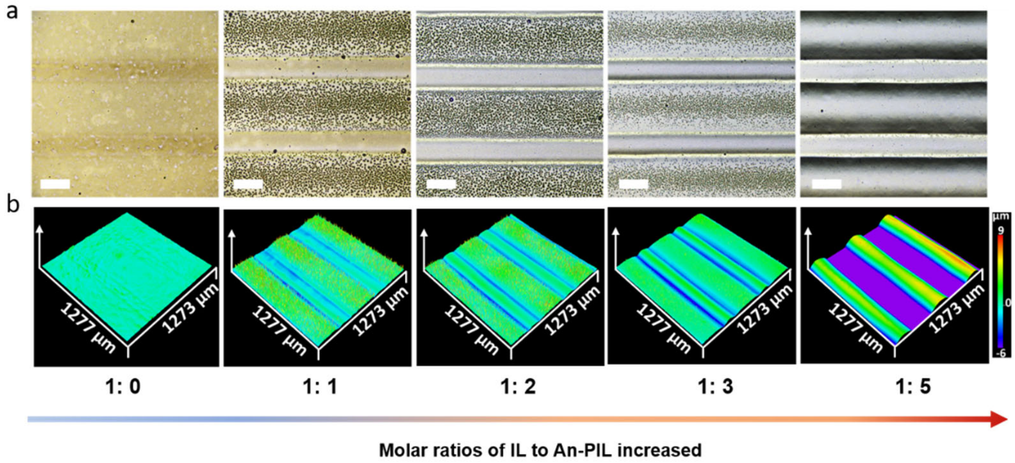

Supplementary Fig. 4. a Regulation of IL droplets secretion on primary patterns with different molar ratios of An-PIL to IL, scale bar:200 μm. **b** The corresponding LSCM images of IL droplets @primary patterns.

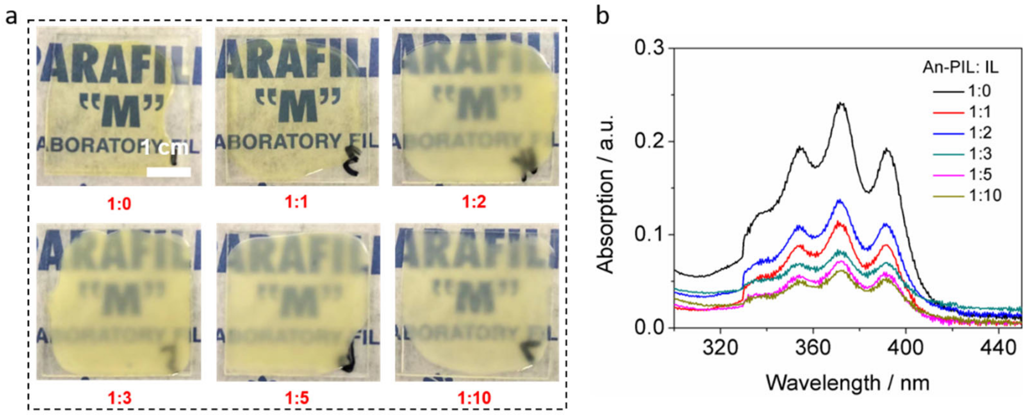

Supplementary Fig. 5. a Optical images of pristine iontronic host with different molar ratios of An-PIL to IL, and **b** The corresponding UV-vis spectra. As the molar ratios of IL to An-PIL increased within iontronic host, the transparency and the UV-vis absorption of the dropping coated films decreased.

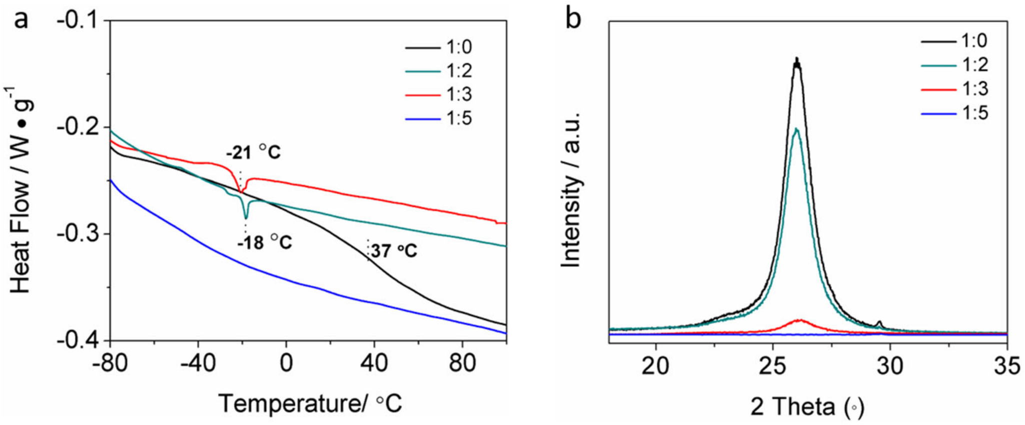

Supplementary Fig. 6. a DSC results and **b** XRD spectra for as-prepared iontronic host with different molar ratios of An-PIL to IL were 1:0 (black line), 1:2 (dark cyan line), 1:3 (red line), and 1:5 (blue line) respectively. As the mole ratios of IL increased, the glass transition temperature (T_g) of the iontronic host decreased from the initial of 37 °C to that of -21°C. Moreover, the intensity of the XRD peak at 26° also decreased as the molar ratios of IL to An-PIL increased.

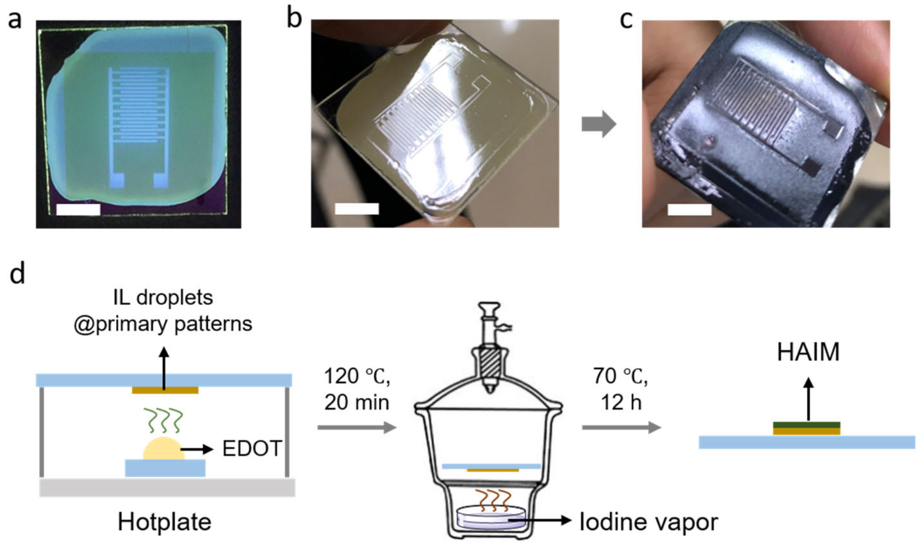

Supplementary Fig. 7. Vapor oxidative polymerization of EDOT upon IL droplets @primary patterns. **a** fluorescence image of pristine IL droplets @primary patterns, **b** the corresponding optical image of **a**, and **c** PEDOT produced upon the IL droplets @primary patterns; scale bar:1 cm. **d** The schematic of the vapor oxidative polymerization of EDOT upon IL droplets @primary patterns.

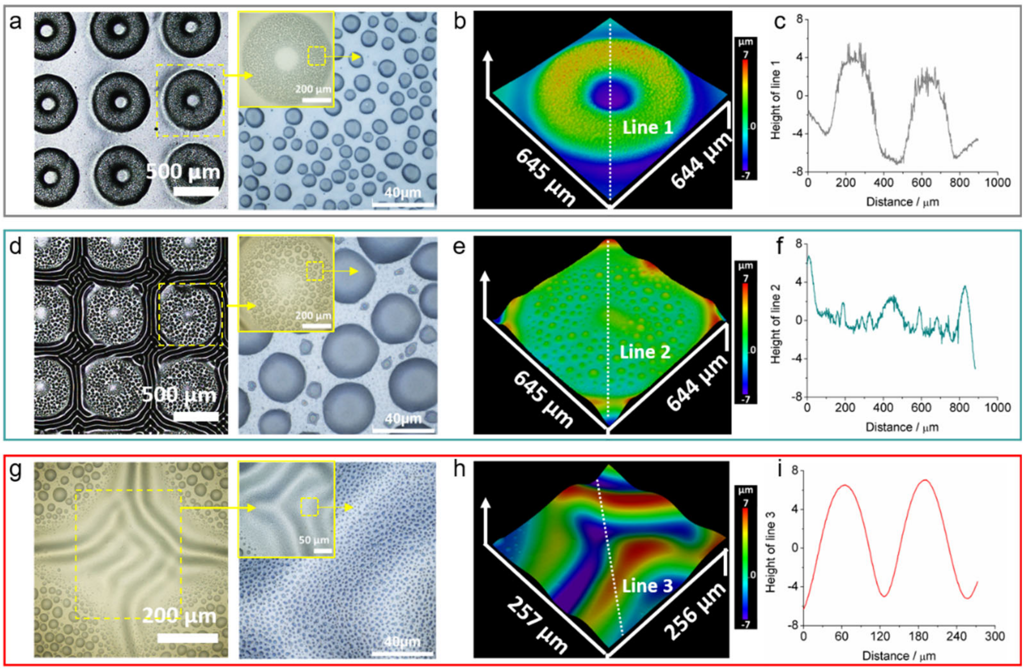

Supplementary Fig. 8. a Optical microscope images, **b** LSCM images, and **c** the profiles of pristine IL droplets @primary patterns. **d** Optical microscope images, **e** LSCM images, and **f** the profiles of pristine IL droplets @primary patterns after absorption of EDOT vapor at 120 °C for 20 min. **g** Optical microscope images, **h** LSCM images, and **i** the profiles of pristine secondary wrinkles.

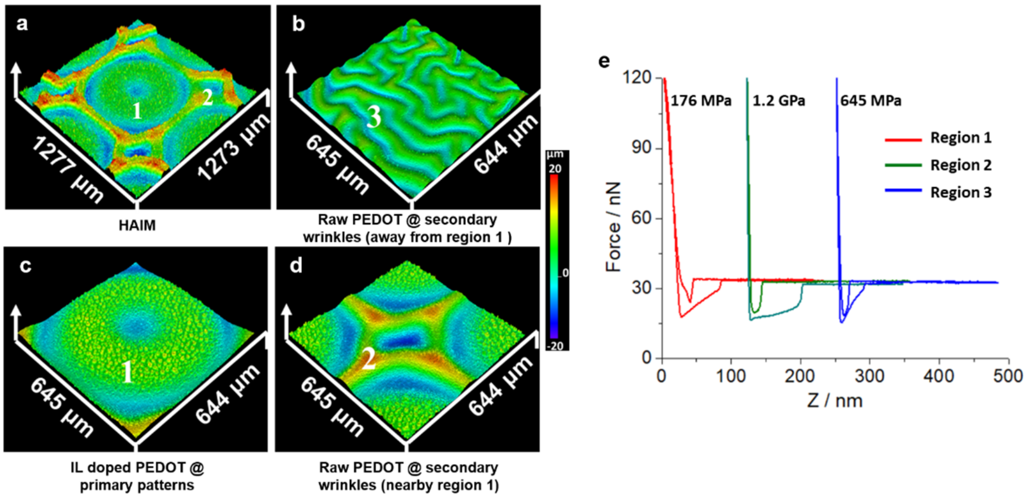

Supplementary Fig. 9. LSCM images of **a** resultant HAIM with different regionally numbered, **b** raw PEDOT @secondary wrinkles away from regions 1, **c** IL doped PEDOT @primary patterns (i.e. corresponding to region 1 in **a**), and **d** raw PEDOT @secondary wrinkles nearby region 1 (i.e. corresponding to region 2 in **a**). **e** Typical force-distance curves recorded on the surfaces of HAIM corresponding to different regions in **a**.

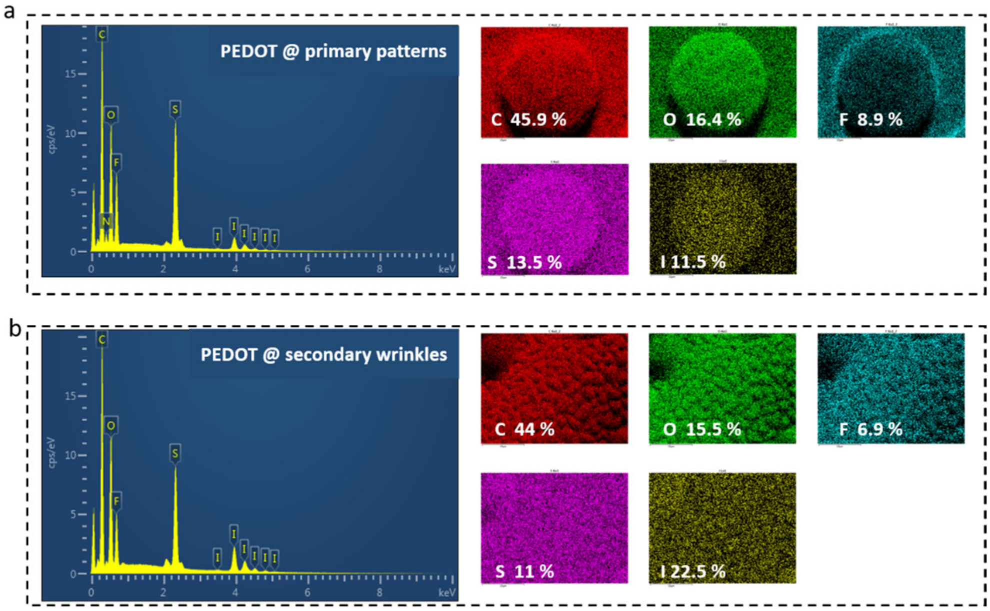

Supplementary Fig. 10. Energy Dispersive Spectrometer (EDS) results and the corresponding elements mapping of **a** PEDOT @ primary patterns and **b** PEDOT @ secondary wrinkles, respectively.

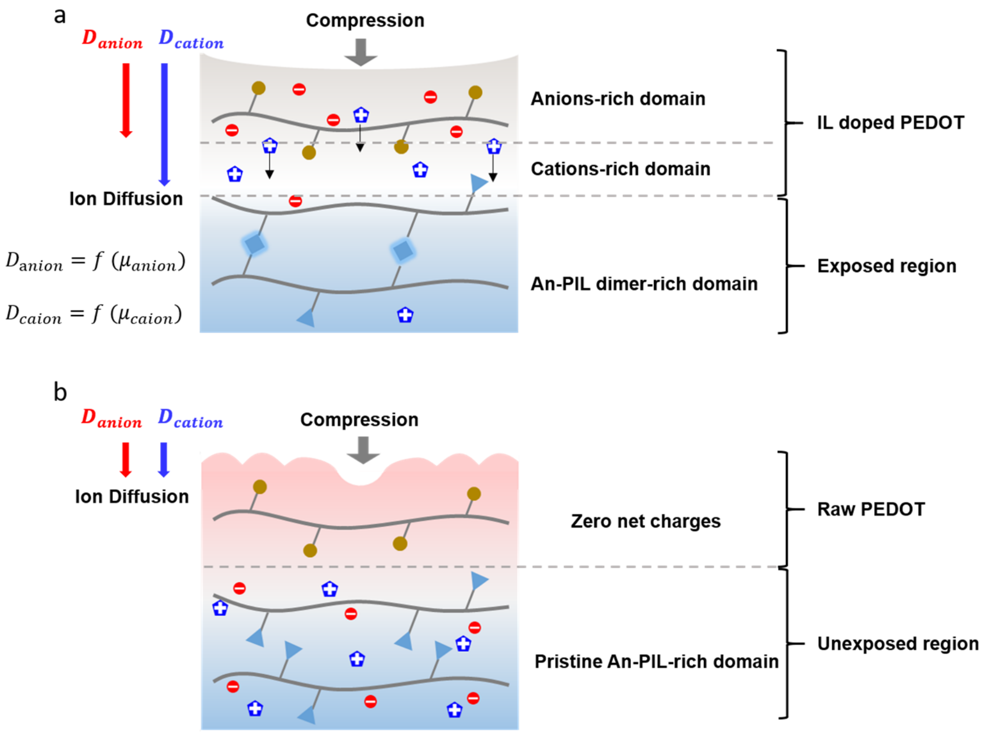

Supplementary Fig. 11. A diagram of Mathematic derivation for the compression-to-potential gradients. The ionic diffusion under compression of **a** the raw PEDOT @primary patterns, and **b** IL-doped PEDOT @secondary wrinkles.

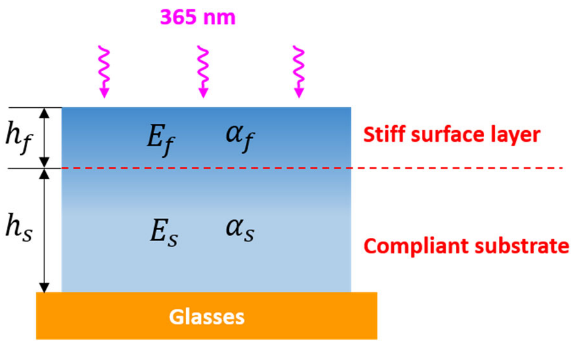

Supplementary Fig. 12. Modified bilayer model of gradient crosslinking layer for iontronic host.

References

1. Mirza S*, et al.* Transparent and conformal'piezoionic'touch sensor. In: *Electroactive Polymer Actuators and Devices (EAPAD) 2015*). SPIE (2015).
2. Dobashi Y*, et al.* Piezoionic mechanoreceptors: Force-induced current generation in hydrogels. *Science* **376**, 502-507 (2022).
3. Xia M*, et al.* Self-Powered Multifunction Ionic Skins Based on Gradient Polyelectrolyte Hydrogels. *ACS nano* **16**, 4714-4725 (2022).
4. Hou H*, et al.* Reversible surface patterning by dynamic crosslink gradients: controlling buckling in 2D. *Adv. Mater.* **30**, 1803463 (2018).
5. Li F, Hou H, Yin J, Jiang X. Near-infrared light–responsive dynamic wrinkle patterns. *Sci. Adv.* **4**, eaar5762 (2018).
